# Autophagy: a decisive process for stemness

**DOI:** 10.18632/oncotarget.7766

**Published:** 2016-02-26

**Authors:** Laura García-Prat, Marta Martínez-Vicente, Pura Muñoz-Cánoves

**Affiliations:** ^1^ Cell Biology Group, Department of Experimental and Health Sciences, Pompeu Fabra University (UPF), CIBER on Neurodegenerative Diseases (CIBERNED), Barcelona, Spain; ^2^ Neurodegenerative Diseases Research Group, Vall d'Hebron Research Institute-CIBERNED, Barcelona, Spain; ^3^ Institució Catalana de Recerca i Estudis Avançats (ICREA), Barcelona, Spain

**Keywords:** autophagy, stem cells, aging, muscle, senescence

## Abstract

Mature skeletal muscle is a stable tissue imposing low homeostatic demand on its stem cells, which remain in a quiescent state in their niche over time. We have shown that these long-lived resting stem cells attenuate proteotoxicity and avoid senescence through basal autophagy. This protective “clean-up” system is lost during aging, resulting in stem cell regenerative decline. Thus, autophagy is required for muscle stem cell homeostasis maintenance.

Muscle stem cells, also called satellite cells (SCs), are essential for skeletal muscle formation and regeneration. In physiological conditions, the major challenge for these cells is maintenance of the quiescent state to preserve their number and functions throughout life [1]. Quiescence confers SCs a “ready-for-action” position to be rapidly activated upon an insult; quiescent cells are also armed with protective gene programs against environmental stresses to maintain homeostasis; yet, little is known about the regulation of the quiescent stem cell state.

Recently, we have demonstrated that young quiescent SCs are equipped with active mechanisms to ensure renovation of intracellular components during homeostasis such as macroautophagy (autophagy hereafter [2]) in order to preserve cell integrity [3]. Autophagy is a process whereby cytoplasmic components (proteins and organelles), after engulfment into autophagosomes, are degraded by the lysosome, constituting an intracellular quality-control mechanism to guarantee cellular homeostasis. Besides the importance of this basal or constitutive autophagy for homeostasis, adaptive or induced autophagy also plays a major role in the response to starvation conditions, by recycling molecules to rebuild new cellular components.

A recent study reported that autophagy provides the nutrients necessary to meet bioenergetic demands during the transition from quiescence to activation [4]. This study proposed a relative lack of nutrient availability during SC activation, which led to the induction of autophagy, mimicking the response to starvation, the best-known fundamental function of autophagy. Instead, our study is the first to demonstrate that constitutive autophagy functions as a cytoprotective and cellular quality control mechanism to balance protein and organelle turnover in homeostatic conditions, which is essential to maintain both the population of SCs and their functional properties.

Quiescence is known to progressively decline with aging due to alterations in circulating/niche and cell-intrinsic factors [1]. Previous studies of our group shed light on the mechanisms regulating SC aging by showing that in sarcopenic muscle of very old, geriatric mice, the normal stem cell quiescent state is substituted by an irreversible pre-senescent state, which results in numerical and functional muscle stem cell decline [5]. The mechanisms accounting for maintenance of quiescence, preservation of the stem cell pool and prevention of senescence during an individual's life remain largely unknown.

It is widely accepted that the activity of self-degrading and recycling cellular processes declines with aging in most organisms. In skeletal muscle, autophagy appears necessary to maintain myofiber integrity, since autophagy impairment leads to neuromuscular junction degeneration and precocious aging [6]. Consistently, our study demonstrates that autophagy is altered in aged SCs. In particular, these cells exhibit defective autophagosome clearance as a consequence of decreased proteolytic activity of lysosomes and/or inability to fuse with autophagosomes. This results in an accumulation of toxic waste (mainly altered proteins and organelles) that impacts negatively on cellular functions. Thus, autophagy is necessary to maintain muscle stem cell homeostasis. Of note, autophagy is alo required to prevent cell death in neurons. Because of their postmitotic state, these cells cannot divide and use basal autophagic flux to remain free of aggregates and damaged intracellular components and avoid neurodegeneration.

At variance with these findings, an elegant study by the Passegué lab demonstrated that stress-induced autophagy by cytokine removal protects hematopoietic stem cells (HSCs) from apoptosis and, importantly, that the autophagic activity does not decline in HSCs with aging [7]. Instead, autophagy is induced in old compared to young cells in basal conditions, and this can be attributed to untoward effects of the aged bone marrow environment. Thus, differently to HSCs, our results demonstrate that in adult life active basal autophagy in SCs prevents senescence entry, thereby preserving cells’ health and function. Consistent with this, inhibition of autophagy by Atg7 gene deletion in young quiescent SCs caused waste accumulation and senescence entry, resulting in stem cell exhaustion and defective muscle regeneration, hence resembling aged stem cells. Conversely, restoring autophagy pharmacologically or genetically reversed senescence and improved regenerative functions of aged muscle stem cells.

Our results further show that autophagy failure in aged SCs and in young Atg7-deficient SCs leads to accumulation of subcellular organelles, including mitochondria. The increased number of dysfunctional mitochondria, caused by impaired mitophagic flux, generates enhanced levels of reactive oxygen species (ROS), which suffice to induce senescence in SCs. The senescence-inducing mechanisms are not completely understood, but may result from ROS-induced DNA damage, as shown in HSCs under oxidative stress, consistently with the accumulation of DNA damage in geriatric and Atg7-deficient SCs. We have also uncovered ROS as a key epigenetic regulator of polycomb repressive complex-1 (PRC1)-dependent expression of the senescence-promoting gene p16^INK4a^ in aging SCs. Indeed, treatment of geriatric and Atg7-deficient mice with antioxidants not only restored PRC1-mediated INK4a repression and prevented senescence entry, but also restored the regenerative capacity of autophagy-dysfunctional SCs after transplantation.

Taken together, quiescence protects stemness through active autophagy in SCs, and loss of autophagy with aging accounts for damaged mitochondria accumulation and increased ROS levels and consequent senescence entry. Further identification of molecular pathways implicated in stemness maintenance and dysregulation in aging and disease will have important implications in stem cell-based regenerative medicine.

**Figure 1:**
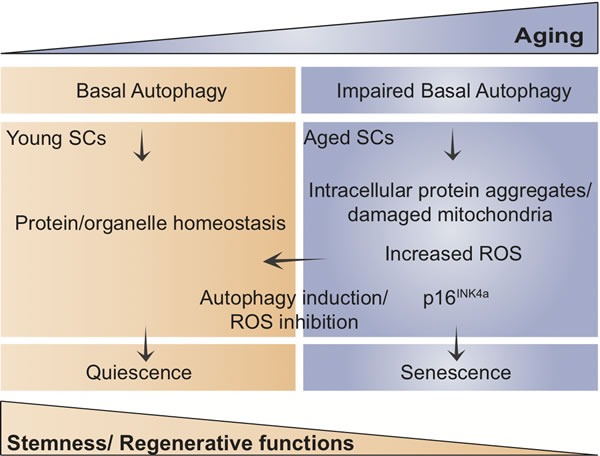
Scheme showing the proposed model of how age-impaired autophagy leads to muscle stem cell senescence and regenerative decline
